# Structural, Functional, and Metabolic Brain Markers Differentiate Collision versus Contact and Non-Contact Athletes

**DOI:** 10.3389/fneur.2017.00390

**Published:** 2017-08-22

**Authors:** Nathan W. Churchill, Michael G. Hutchison, Alex P. Di Battista, Simon J. Graham, Tom A. Schweizer

**Affiliations:** ^1^Neuroscience Research Program, St. Michael’s Hospital, Toronto, ON, Canada; ^2^Keenan Research Centre for Biomedical Science of St. Michael’s Hospital, Toronto, ON, Canada; ^3^Faculty of Kinesiology and Physical Education, University of Toronto, Toronto, ON, Canada; ^4^Institute of Medical Science, University of Toronto, Toronto, ON, Canada; ^5^Department of Medical Biophysics, University of Toronto, Toronto, ON, Canada; ^6^Physical Sciences Platform, Sunnybrook Research Institute, Toronto, ON, Canada; ^7^Faculty of Medicine (Neurosurgery), University of Toronto, Toronto, ON, Canada; ^8^The Institute of Biomaterials & Biomedical Engineering (IBBME) at the University of Toronto, Toronto, ON, Canada

**Keywords:** concussion, subconcussive, contact sports, diffusion tensor imaging, magnetic resonance spectroscopy, functional magnetic resonance imaging

## Abstract

There is growing concern about how participation in contact sports affects the brain. Retrospective evidence suggests that contact sports are associated with long-term negative health outcomes. However, much of the research to date has focused on former athletes with significant health problems. Less is known about the health of current athletes in contact and collision sports who have not reported significant medical issues. In this cross-sectional study, advanced magnetic resonance imaging (MRI) was used to evaluate multiple aspects of brain physiology in three groups of athletes participating in non-contact sports (*N* = 20), contact sports (*N* = 22), and collision sports (*N* = 23). Diffusion tensor imaging was used to assess white matter microstructure based on measures of fractional anisotropy (FA) and mean diffusivity (MD); resting-state functional MRI was used to evaluate global functional connectivity; single-voxel spectroscopy was used to compare ratios of neural metabolites, including *N*-acetyl aspartate (NAA), creatine (Cr), choline, and myo-inositol. Multivariate analysis revealed structural, functional, and metabolic measures that reliably differentiated between sport groups. The collision group had significantly elevated FA and reduced MD in white matter, compared to both contact and non-contact groups. In contrast, the collision group showed significant reductions in functional connectivity and the NAA/Cr metabolite ratio, relative to only the non-contact group, while the contact group overlapped with both non-contact and collision groups. For brain regions associated with contact sport participation, athletes with a history of concussion also showed greater alterations in FA and functional connectivity, indicating a potential cumulative effect of both contact exposure and concussion history on brain physiology. These findings indicate persistent differences in brain physiology for athletes participating in contact and collision sports, which should be considered in future studies of concussion and subconcussive impacts.

## Introduction

While the majority of athletes who participate in collision and contact sports do not present with debilitating clinical outcomes, there is a growing concern for the long-term brain health of these athletes. These concerns largely stem from studies that have identified elevated risk of depression, memory problems, cognitive impairments, and earlier onset of Alzheimer’s disease among former professional football players (1–3). In addition, there is literature linking repetitive head impacts to neurodegeneration and chronic traumatic encephalopathy (CTE) (4, 5), although the etiology of CTE is the subject of ongoing debate. To date, there has been limited research examining the brain health of athletes who are actively participating in contact sports. These athletes may be exposed to hundreds of impacts over a single season (6, 7) and may be at risk of cumulative effects caused by repeated subconcussive blows (8). However, there have been inconsistent findings regarding the negative short-term cognitive effects of athletes exposed to repetitive head impacts over a single season compared to non-contact athletes (9–12). Evaluating the brain physiology of current contact and non-contact athletes may therefore help improve our understanding of the etiology underlying potential long-term health consequences of contact exposure.

Magnetic resonance imaging (MRI) is a non-invasive technique that is capable of measuring alterations in the brain structure, function, and neural metabolite concentrations. In recent years, several studies have used MRI sequences to demonstrate longitudinal changes in neurobiology associated with subconcussive impacts. Chun et al. (13) used diffusion tensor imaging (DTI) to measure the microstructure of white matter in a group of high school football players, showing a correlation between white matter abnormalities and exposure to head impacts. Similarly, another study by Abbas et al. (14) used functional MRI (fMRI) to evaluate functional connectivity of the resting brain for football players over a single season, showing increased functional connectivity between brain regions associated with the default mode network (DMN) (15). In alignment with these findings, Johnson et al. (16) also reported altered functional connectivity of the DMN for rugby players following participation in a full-contact game. In addition, Poole et al. (17) used single-voxel spectroscopy (SVS) to quantify metabolite levels in the brains of football players before and after a competitive season, showing significant deviations from baseline at the end of the season. Collectively, these results demonstrate significant variations in the brain structure, function, and neural metabolite levels among contact sport participants within a single game or over the course of a season.

To date, studies have primarily focused on male, American football players spanning a single season. Much less is known about MRI measures of brain structure and function associated with sport participation across the wider sporting community, which encompasses male and female athletes at different levels of contact exposure. In addition, prior studies examining contact sports have focused exclusively on a single MRI parameter, providing limited information about the relationship between the different MRI measures of brain structure and function. As defined by Meehan et al. (18), sports may be categorized into three subsets based on the level of contact exposure: non-contact, contact (body-to-body contact allowed, but not purposeful collisions), or collision (routine, purposeful body-to-body collisions). It is currently unknown whether there are consistent differences in brain structure, function, and neural metabolites associated with participation in these different sport categories.

In the present study, these gaps in knowledge are addressed by providing a comprehensive examination of MRI measures of white matter microstructure (DTI), resting brain function (fMRI) and neural metabolites (SVS). Three groups of athletes (i.e., drawn from non-contact, contact and collision sports) were examined preseason, to determine whether there are persistent “baseline” neurobiological differences between groups. This has significant implications for neuroimaging of athletes, by establishing whether there are persistent markers of contact exposure which are not attributable to recent concussive or subconcussive impacts acquired during competitive play. This study employed a representative sample of the currently active athlete population, which included healthy athletes who do not present with significant clinical impairments. Each MRI measure was tested for reliable differences between groups using a flexible multivariate modeling approach. An additional set of analyses examined whether brain regions that showed significant effects of contact exposure were also affected by prior concussion history.

## Materials and Methods

### Participants

Sixty-five athletes were recruited from interuniversity (“varsity”) teams at a single institution *via* the Sport Medicine Clinic, including 20 athletes from non-contact sports, 22 athletes from contact sports, and 23 from collision sports. Athletes were approached during preseason baseline testing and were reimbursed for their time with a token monetary compensation, in accordance with Research Ethics Board (REB) guidelines. Imaging was conducted at the start of their respective competitive seasons to focus on persistent markers of contact sport participation, control for transient physiological effects during competition and minimize exposure to recent subconcussive impacts. Athletes were matched across groups on sex and prior concussion history, both of which have been previously identified as risk modifiers for concussion (19). Participant demographics and sport representations are listed in Table 1. All athletes were required to have sustained no concussions within the 6 months prior to imaging; for athletes with a history of concussion, the median time since their last injury was 2 years (range: 9 months to 8 years). Preseason assessments were also conducted in-clinic using the Sport Concussion Assessment Tool 3 (SCAT3) (20) to evaluate symptoms, cognitive function, and balance. This study was carried out in accordance with the recommendations of the Canadian Tri-Council Policy Statement 2 and the REBs of the University of Toronto and St. Michael’s Hospital, with written informed consent from all participants. All participants gave written informed consent in accordance with the Declaration of Helsinki. The protocol was approved by the REBs of the University of Toronto and St. Michael’s Hospital.

**Table 1:** Study demographics.

	Non-contact	Contact	Collision
Age	20.0 ± 1.7	20.3 ± 1.5	21.3 ± 1.9
Female	10/20	14/22	9/23
Prior concussion	8/20	9/22	11/23
Sport	Volleyball (20)	Soccer (11)	Rugby (9)
		Field hockey (5)	Ice hockey (8)
		Basketball (4)	Lacrosse M (3)
		Lacrosse F (1)	Football (3)
		Water polo (1)	
**Symptom severity**			
Total	1 [0, 29]	5 [0, 17]	2 [0, 20]
Somatic	0 [0, 17]	1 [0, 8]	1 [0, 7]*
Cognitive	0 [0, 7]	0 [0, 5]	0 [0, 5]
Sleep/fatigue	0 [0, 4]	2 [0, 7]	0 [0, 7]
Emotional	0 [0, 3]	0 [0, 5]	0 [0, 3]
**Cognitive testing**			
Orientation	5 [4, 5]	5 [4, 5]	5 [4, 5]
Immediate memory	15 [14, 15]	15 [10, 15]	15 [9, 15]
Concentration	4 [1, 5]	3 [1, 5]	3 [1, 5]
Delayed recall	4 [0, 5]	4 [1, 5]	4 [1, 5]
Total balance score	1 [0, 10]	2 [0, 10]	3 [0, 9]

*Symptoms severity scores are based on Sport Concussion Assessment Tool 3 assessment and are based on the total of Likert scores, summed across all relevant symptom subscales. Age is reported as mean ± SD, and clinical scores are reported as median [minimum, maximum]*.

*Clinical scale where contact or collision sport is significantly higher than non-contact at *p* < 0.05 (uncorrected), based on non-parametric Mann–Whitney test.

### Magnetic Resonance Imaging

Participants were imaged at St. Michael’s Hospital using an MRI system operating at 3 T (Magnetom Skyra, Siemens, Erlangen, Germany) and standard 20-channel head receiver coil. Multimodal MRI was acquired for all participants, with analysis and preprocessing details provided below.

#### Anatomical Imaging

To perform between-subject alignment of fMRI data, T1-weighted MPRAGE was obtained, with field of view (FOV) = 24 cm × 24 cm, 240 × 240 × 192 acquisition matrix, 0.9 mm isotropic voxels, bandwidth = 250 Hz/Pixel, inversion time (TI)/echo time (TE)/repetition time (TR) = 850/2.63/2,000 ms, and flip angle = 8^o^. In addition, FLAIR was obtained to assess for structural lesions, with FOV = 22 cm × 18.6 cm, 256 × 196 acquisition matrix, 1.1 mm × 0.9 mm × 3.0 mm voxels, TI/TE/TR = 2,200/96/9,000 ms. Susceptibility-weighted imaging was also acquired to assess for vascular abnormalities, with 220 × 192 FOV, 0.6 mm × 0.6 mm × 1.2 mm voxels. TE/TR = 20/28 ms, flip angle = 15°, 384 × 307 with encoding gap of 0.2 mm.

#### White Matter Microstructure

Diffusion-weighted imaging was acquired with 30 encoding directions (*b* = 700 s/mm^2^, FOV = 24 cm × 24 cm, 120 × 120 acquisition matrix, 66 axial slices, 2 mm isotropic voxels, TE/TR = 83/7,800 ms, bandwidth = 1,736 Hz/Px). The FSL^1^ *eddy_correct* protocol was used to perform simultaneous correction of eddy currents and rigid-body motion correction, *bet* was used to mask out non-brain voxels, and *dtifit* was used to calculate voxelwise measures of fractional anisotropy (FA), which reflects the directionality of water diffusion in white matter tracts and mean diffusivity (MD), which quantifies the total amount of water diffusion, independent of direction. The individual subject FA and MD maps were co-registered to a common template space, based on the FSL FDT protocol: (1) individual masked FA maps were eroded by 1 voxel width at brain edges and co-registered to the FMRIB58 template (1 mm^3^) *via* affine transform, using flirt; (2) a symmetric, study-specific template was computed by averaging transformed FA maps and then re-averaging with flipped left/right orientations; (3) the average template was used as a reference and non-linear registration of FA maps performed using *fnirt*, which were used to update the study-specific template; and (4) the FA maps were registered to the new template via *fnirt* and the mean template was updated again. During the final registration step, images were resampled to 2 mm^3^ resolution. Prior to analysis, images were also convolved with a 6-mm full width at half maximum (FWHM) 3D Gaussian smoothing kernel, to minimize the effects of local variations in white matter structure. The analyses were performed within a mask of regions with a mean FA > 0.20, to restrict analyses to probable white matter tracts. The brainstem and cerebellum were also manually segmented and removed, to avoid confounding effects of spatial registration errors caused by significant magnetic field inhomogeneity in these regions. Subsequent analyses compared FA and MD brain maps between the sport groups.

#### Brain Function

Resting-state fMRI was acquired *via* multi-slice T2*-weighted echo planar imaging (FOV = 20 cm × 20 cm, 64 × 64 matrix, 32 slices, 3.125 mm × 3.125 mm × 4.5 mm voxels, TE/TR = 30/2,000 ms, flip angle = 70°, and oblique axial interleaved), producing a time series of 194 images. During acquisition, participants were instructed to lie still with their eyes closed and not focus on anything in particular. Processing and analysis were performed using the Analysis of Functional Neuroimages (AFNI) package^2^ and customized algorithms developed in the laboratory. This included rigid-body motion correction (AFNI *3dvolreg*), removal of outlier scan volumes using the SPIKECOR algorithm,^3^ slice-timing correction (AFNI *3dTshift*), spatial smoothing with a 6-mm FWHM isotropic 3D Gaussian kernel (AFNI *3dmerge*), and regression of motion parameters and linear-quadratic trends as nuisance covariates. To control for physiological noise, data-driven correction was performed using the PHYCAA + algorithm,^4^ along with regression of white matter signal, using the FSL *fast* algorithm to segment the T1 anatomical scan and regress out mean signal in white matter voxels (*p* > 0.95). The fMRI data were coregistered into a common template space by computing the rigid-body transform of the mean fMRI volume for each participant to their T1-weighted anatomical image and the 12-parameter affine transformation of the T1 image for each participant to the MNI152 template. The transformation matrices were concatenated and the net transform applied to fMRI data, resampled at 2-mm^3^ resolution. Global functional connectivity (Gconn) was then estimated for each voxel, by computing the functional connectivity with all other brain voxels, as the Pearson correlation between fMRI time series. Gconn was measured as the mean of all (positive) connectivity values, providing a voxelwise measure of total integrative function. Subsequent analyses compared the Gconn brain maps between the different sport groups.

#### Neural Metabolites

Single-voxel ^1^H spectroscopy data were acquired for two regions of interest, placed on left and right hand motor knobs. This was obtained *via* stimulated echo acquisition mode (STEAM) for 2 cm isotropic voxels (TM/TE/TR = 10/30/2,000 ms; bandwidth = 1,200 Hz; FA = 40°; 100 acquisitions; 1,024 points). Regions were placed on an AC-PC-oriented axial slice corresponding to the region of interest first and confirmed using coronal and axial views to ensure adequate distance from ventricles, fatty tissue, and bone. Processing and analysis was conducted using the TARQUIN software package^5^ with default preprocessing parameter settings for STEAM, to obtain relative metabolite concentration values. The following metabolites were then analyzed: *N*-acetyl aspartate (NAA), choline (Cho), creatine (Cr), and myo-inositol (Ins). All six unique pairwise ratios of the different metabolites were compared between sport groups (NAA/Cr, NAA/Cho, Ins/Cr, Ins/Cho, Cho/Cr, and NAA/Ins), after averaging ratio values across left- and right-side motor cortex.

### Statistical Analyses

All statistical analyses were conducted in MATLAB. An initial set of analyses compared the symptom score profiles for the non-contact, contact and collision groups. The SCAT3 symptom severity scores were based on a 22-item symptom scale, each assessed on a 7-point Likert scale. Non-parametric Mann-Whitney tests were used to compare SCAT3 total severity (summed over all symptom subscales), along with cognitive and balance error scores for athletes in contact and collision sports, relative to non-contact athletes. In addition, total severity was compared for four specific symptom clusters: somatic (headache, pressure in head, neck pain, nausea/vomiting, dizziness, blurred vision, balance problems, sensitivity to light, and sensitivity to noise), cognitive (feeling slowed down, feeling “in a fog,” “don’t feel right,” difficulty concentrating, difficulty remembering, confusion), fatigue and sleep problems (fatigue/low energy, drowsiness, trouble falling asleep), and emotional (more emotional, irritability, sadness, nervous/anxious). Correction for multiple comparisons was subsequently performed across all subscales, at a false-discovery rate (FDR) of 0.05.

Multivariate partial least squares (PLS) analysis was performed for each MRI measure (FA, MD, Gconn, and metabolite ratios). This widely used linear latent variable model identifies brain regions that show significant covariation across groups (21–23). Mean-centered task PLS was used to identify patterns of brain voxels (FA, MD, and Gconn) or metabolite ratios that show covariation across the three sport groups (non-contact, contact, and collision). Each task PLS analysis produced a pattern of “voxel” (or “metabolite”) saliences, reflecting the importance of specific brain regions (or metabolite ratios), and a set of “group” saliences, reflecting how much each group expressed the associated brain pattern. The first PLS component is reported for each MRI modality, which explains the greatest amount of data covariance.

The significance of PLS saliences was evaluated *via* bootstrap resampling (1,000 iterations for each analysis). The significance of voxel (or metabolite) saliences was expressed as the bootstrap ratio of (mean/SE) for each variable. For FA, MD, and Gconn, correction for multiple comparisons was obtained by applying a voxel-level significance threshold of *p* < 0.005, followed by cluster-size thresholding using AFNI (see text footnote 2) program *3dFWHMx* to estimate spatial smoothness and *3dClustSim* to identify the minimum cluster size at an adjusted *p* = 0.05 threshold. Thresholded brain maps are shown as maximum intensity projections in each imaging plane, centered on the MNI space coordinates (*x* = 8, *y* = −14, and *z* = 6). For SVS, correction for multiple comparisons was performed at an FDR of 0.05. *Post hoc* bootstrapped *p* values were computed on the difference in group saliences between each pair of sport groups, also corrected at an FDR of 0.05. Finally, the distribution of subject MRI values was plotted for each sport group, by computing the average over all significant voxels (or metabolite ratios) for each athlete.

For each MRI measure, further analysis was performed to determine whether the brain regions (or metabolite ratios) showing significant variation across sport groups were also affected by concussion history. The mean value was computed within significant brain regions for each subject, and then the mean difference was computed between all athletes with a history of concussion (*n* = 27) relative to those without prior concussion (*n* = 37). Bootstrap resampling was then performed to obtain the 95% confidence interval (CI) on mean differences, along with the empirical *p* value, under the one-tailed hypothesis that concussion shows a similar direction of effect as contact exposure. For MRI measures showing a significant relationship, an additional set of *post hoc* analyses tested for differences associated with concussion history within individual sport groups (non-contact, contact, and collision), to determine whether a specific sport group showed greater effects of concussion history.

## Results

### Clinical Data

Table 1 summarizes the demographic information for each of the three athlete groups. There were no significant between-group differences for sex and prior concussion history (*p* ≥ 0.11 uncorrected for all comparisons, Mann–Whitney tests). Examining preseason symptom scores, total symptom severity was not significantly different between groups (*p* ≥ 0.18, all pairwise comparisons). Only somatic complaints showed a significant difference between groups, with higher scores for collision sports compared to non-contact sports (mean difference ± SE: 1.0 ± 0.5; *p* = 0.017), although the effect was non-significant after adjusting for multiple comparisons at an FDR of 0.05. Cognitive and balance scores also showed no significant differences between groups (*p* ≥ 0.26, all pairwise comparisons). These results indicate a general absence of significant clinical differences between the sport groups in this study.

### Neuroimaging Data

**Figure 1:**
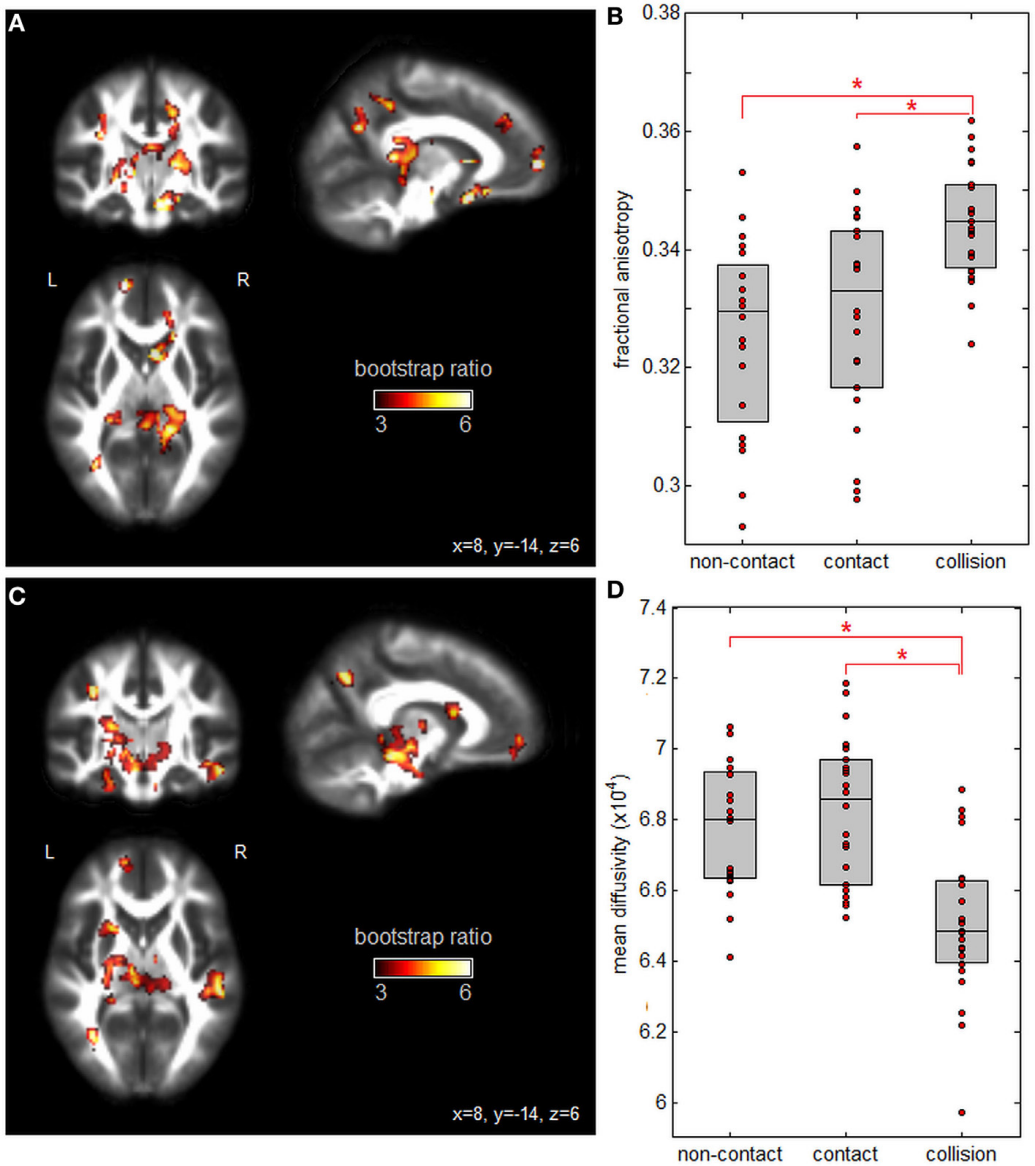
Effects of sport contact level on fractional anisotropy (FA) and mean diffusivity (MD) of white matter. **(A)** Brain regions showing reliable differences in FA between sport groups, overlaid on FMRIB58_FA diffusion tensor imaging atlas. **(B)** Mean subject FA values per group, with upper and lower distribution quartiles (gray boxes). **(C)** Brain regions showing reliable differences in MD between sport groups and **(D)** mean subject MD values per group, with upper and lower distribution quartiles. *Significant differences between groups at a false-discovery rate of 0.05. Thresholded brain maps are shown as maximum intensity projections in each imaging plane, centered on the MNI space coordinates (*x* = 8, *y* = −14, and *z* = 6).

Figure 1 plots PLS analysis results for the DTI measures of FA and MD. Figure 1A depicts voxel saliences for FA, where a set of white matter clusters showed significant positive bootstrap ratios, indicating reliable differences in FA values across groups (see Table 2 for cluster report). This included clusters in the splenium of the corpus callosum, fornix, internal and external capsules, and corona radiata. Figure 1B depicts the distribution of mean subject FA values averaged over significant brain regions, for each group. Athletes in collision sports had significantly higher FA than non-contact and contact sports (*p* = 0.004 and *p* < 0.001, respectively, significant at FDR = 0.05), while non-contact and contact groups were not significantly different (*p* = 0.21). Figure 1C shows voxel saliences for MD, where a set of significant clusters also indicate reliable differences in MD across groups (see Table 3 for cluster report). Similar to FA, significant effects were seen in the corona radiata and internal capsule; however, distinct clusters were also seen in the cerebral peduncle, sagittal striatum, and hippocampus. Figure 1D plots the distribution of mean subject MD values, averaged over all significant brain regions, for each sport group. The trend was the opposite of FA, where athletes in collision sports had significantly lower MD than non-contact and contact sports (*p* = 0.002 and *p* < 0.001, respectively, significant at FDR = 0.05), while non-contact and contact were not significantly different (*p* = 0.42).

**Table 2:** Cluster report for fractional anisotropy (FA), showing all clusters significant at an adjusted *p* < 0.05.

Cluster	Center of mass			Brain region	Cluster size (mm^3^)	Peak value (bootstrap ratio)
1	18	−32	10	Internal capsule (retrolenticular) R	2,152	6.03
2	0	−34	18	Corpus callosum (splenium) R	1,160	4.85
3	10	14	−10	External capsule R	904	6.57
4	−22	−32	2	Fornix (cres) L	720	4.69
5	−30	−60	34	Posterior corona radiata L	640	5.92
6	14	−46	44	Posterior corona radiata R	632	5.04
7	−14	58	6	Anterior corona radiata L	624	7.07
8	16	36	32	Anterior corona radiata R	552	4.59

*The center of mass is given in MNI space coordinates. Brain regions indicate location of the center of mass, based on the John Hopkins University (JHU) white matter atlas*.

**Table 3:** Cluster report for mean diffusivity, showing all clusters significant at an adjusted *p* < 0.05.

Cluster	Center of mass			Brain region	Cluster size (mm^3^)	Peak value (bootstrap ratio)
1	−8	−16	−6	Cerebral peduncle L	3,464	5.33
2	48	−28	−14	Sagittal striatum R	1,912	5.88
3	−24	−24	−18	Cingulum (hippocampus) L	1,032	4.52
4	−24	10	16	Internal capsule (anterior limb) L	1,016	5.04
5	−12	54	−8	Anterior corona radiata L	632	4.81
6	−34	−62	32	Posterior corona radiata L	592	5.77

*The center of mass is given in MNI space coordinates. Brain regions indicate location of the center of mass, based on the John Hopkins University white matter atlas*.

**Figure 2:**
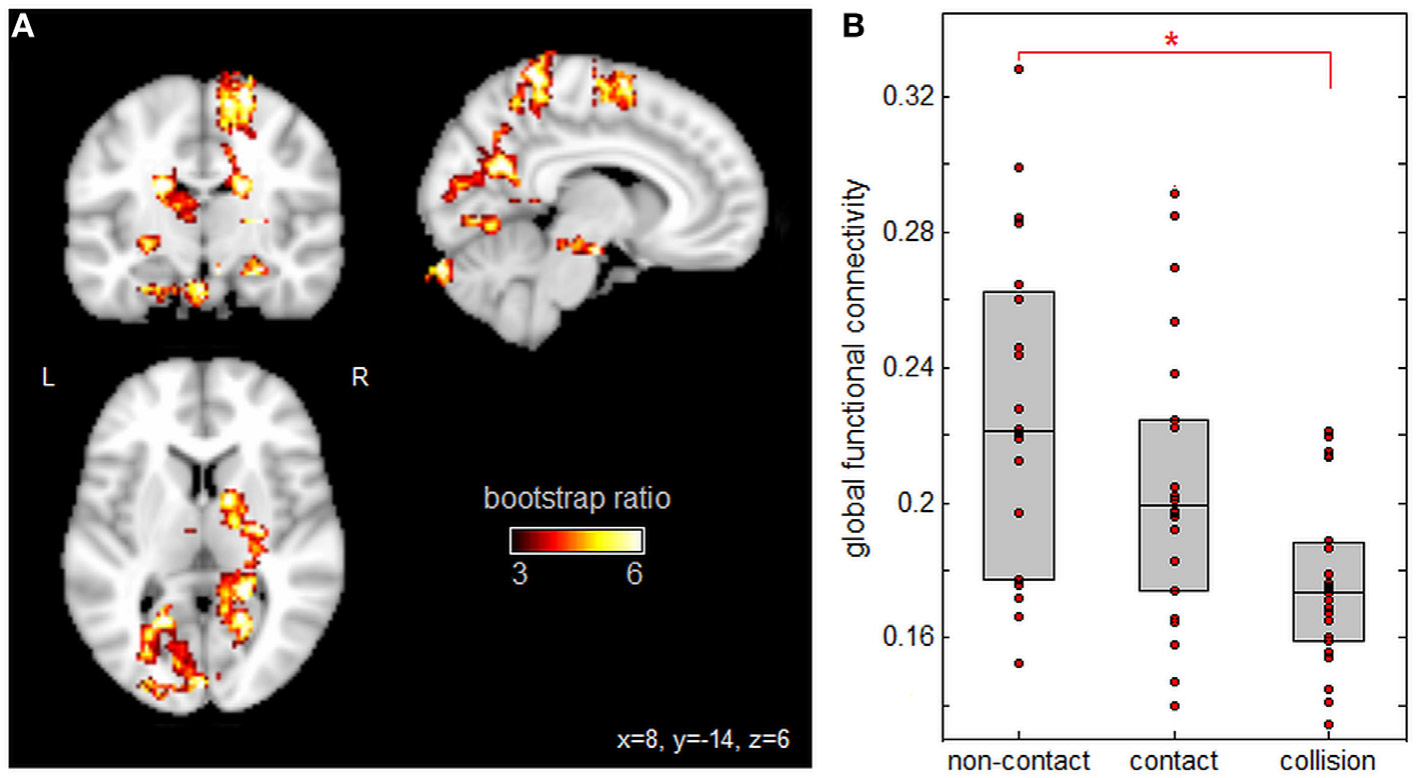
Effects of sport contact level on global functional connectivity (Gconn) of gray matter. **(A)** Brain regions showing reliable differences in Gconn between sport groups, overlaid on MNI152 T1 atlas. **(B)** Mean subject Gconn values per group, with upper and lower distribution quartiles (gray boxes). *Significant differences between groups at a false-discovery rate of 0.05. Thresholded brain maps are shown as maximum intensity projections in each imaging plane, centered on the MNI space coordinates (*x* = 8, *y* = −14, and *z* = 6).

Figure 2 displays PLS analysis results for the Gconn values derived from resting-state fMRI. As shown by the voxel salience map in Figure 2A, a set of brain regions were identified with significant positive bootstrap ratios, indicating reliable differences in Gconn across groups (see Table 4 for cluster report). This included multiple regions implicated in visual processing and attention, including superior parietal lobe, precuneus, superior occipital lobe, calcarine sulcus, and fusiform gyrus. Significant regions were also implicated in motor coordination, including the supplementary motor area and cerebellum. In addition, the hippocampus showed significant effects; this brain region is implicated in non-verbal memory. Figure 2B depicts the distribution of mean subject Gconn values, averaged over significant brain regions, for each sport group. Gconn was highest for athletes in non-contact sports, intermediate for athletes in contact sports, and lowest for athletes in collision sports. However, only collision and non-contact sports were significantly different after correcting for multiple comparisons (*p* < 0.001, significant at an FDR of 0.05), whereas contact sport athletes could not be distinguished from either non-contact or collision sport athletes (*p* ≥ 0.13 for both comparisons).

**Table 4:** Cluster report for global functional connectivity (Gconn), showing all clusters significant at an adjusted *p* < 0.05.

Cluster	Center of mass			Brain region	Cluster size (mm^3^)	Peak value (bootstrap ratio)
1	18	−44	66	Parietal Sup. R	3,136	5.96
2	14	−2	62	Supp. Motor Area R	1,912	5.12
3	18	−60	26	Precuneus R	1,512	5.52
4	−10	−80	14	Calcarine L	992	4.11
5	−8	−90	−30	Cerebellum Crus2. L	920	6.09
6	−18	−60	24	Occipital Sup. L	888	5.55
7	−26	−70	−6	Fusiform L	800	4.69
8	26	−20	−18	Hippocampus R	768	5.05

*The center of mass is given in MNI space coordinates. Brain regions indicate location of the center of mass, based on the Automated Anatomical Labelling (AAL) atlas*.

**Figure 3:**
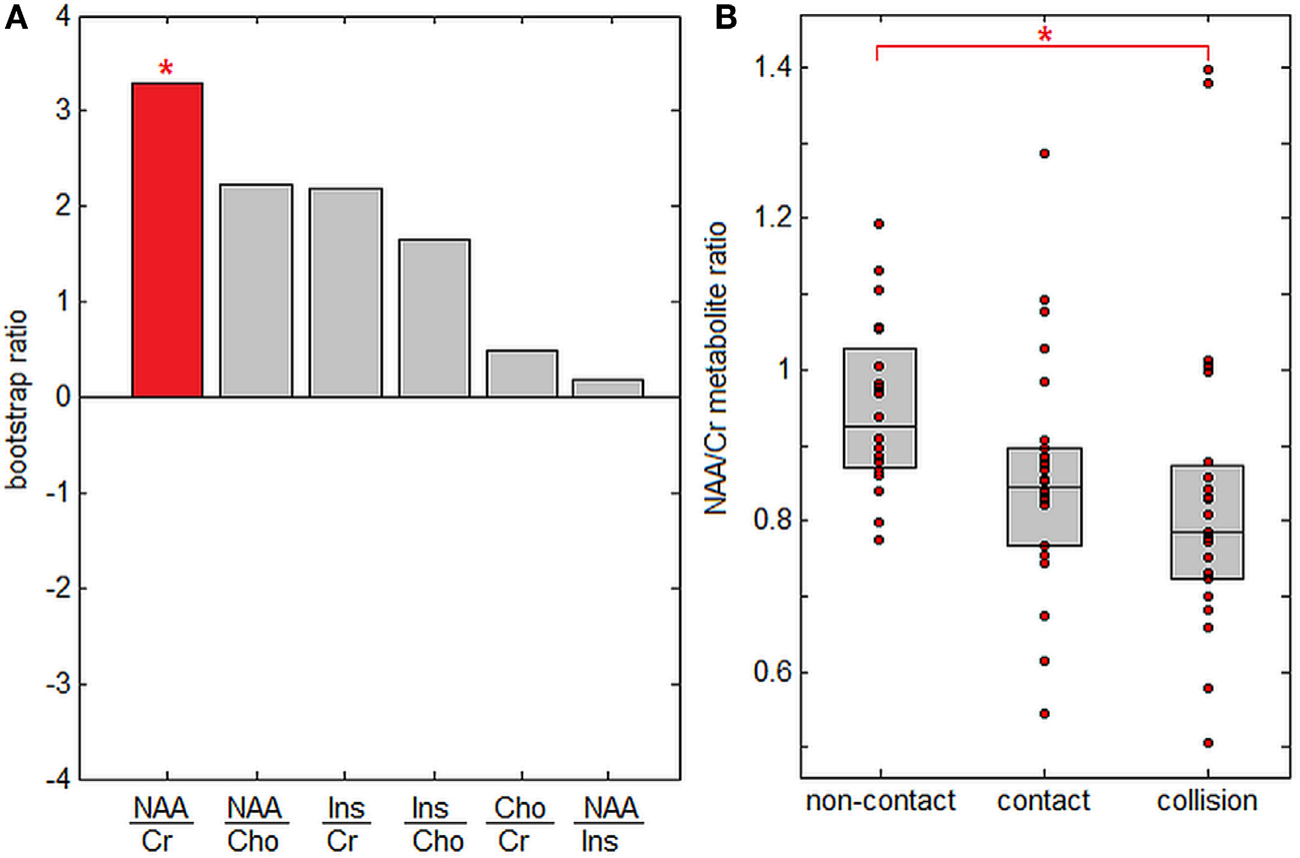
Effects of sport contact level on cerebral metabolites. **(A)** Metabolite ratios showing reliable differences between sport groups. The red bar indicates that *N*-acetyl aspartate to creatine (NAA/Cr) is the only significantly altered metabolite after multiple comparison correction. **(B)** Mean subject NAA/Cr ratios per group, with upper and lower distribution quartiles (gray boxes). *Significant differences between groups at a false-discovery rate of 0.05.

Figure 3 plots PLS analysis results for brain metabolite ratios. In Figure 3A, saliences are plotted for all analyzed metabolite ratios, ordered by effect size. Only the NAA/Cr ratio showed significant between-group differences after correcting for multiple comparisons at FDR = 0.05, although NAA/Cho and Ins/Cho ratios were significant at *p* < 0.05 before correction. Figure 3B plots subject NAA/Cr ratios per group, showing progressive effects, with the highest values for athletes in non-contact sports, intermediate values for athletes in contact sports, and lowest values for athletes in collisions sports. Only the difference in NAA/Cr values between athletes in non-contact and collision sports was significant after correcting for multiple comparisons (*p* < 0.001, significant at FDR = 0.05), while contact sport athletes could not be distinguished from either non-contact or collision sport athletes (*p* ≥ 0.35 for both).

Table 5 reports neuroimaging parameter values for athletes with and without a history of concussion, for each sport group. Combining all sport groups, FA within brain regions implicated in contact exposure showed significant effects of concussion history (mean increase: 0.0069, 95% CI: 0.0187, 0.0007; *p* = 0.029), whereas MD did not (mean decrease: −0.022 × 10^−5^, 95% CI: −1.697 × 10^−5^, 0.983 × 10^−5^; *p* = 0.47). Similarly, Gconn showed significant effects of concussion history (mean decrease: −0.021, 95% CI: 0.004, 0.055; *p* = 0.034), while effects on the NAA/Cr ratio were non-significant (mean decrease: −0.037, 95% CI: −0.174, 0.029; *p* = 0.18). Supplemental analyses of FA and Gconn examined whether specific sport subgroups were more sensitive to the effects of prior concussions. For FA, none of the individual sport subgroups showed a significant association with concussion history (*p* ≥ 0.11 for all), but for Gconn the non-contact group showed significant effects (median decrease: −0.042, 95% CI: 0.007, 0.104; *p* = 0.026), while other sport groups were non-significant (*p* ≥ 0.059 for both).

**Table 5:** Imaging parameter values for each sport group (non-contact, contact, and collision), for athletes with and without history of concussion.

	FA*	Mean diffusivity (MD) (×10^−5^)	Global functional connectivity (Gconn)*	*N*-acetyl aspartate to creatine (NAA/Cr)
Non-contact, no concussion	0.327 [0.293, 0.346]	67.8 [65.0, 70.4]	0.234 [0.142, 0.328]	0.97 [0.78, 1.18]
Non-contact, concussion	0.331 [0.308, 0.354]	67.2 [63.9, 69.5]	0.182 [0.156, 0.283]	0.86 [0.76, 0.97]
Contact, no concussion	0.319 [0.296, 0.356]	69.3 [65.2, 71.6]	0.201 [0.133, 0.296]	0.85 [0.68, 0.91]
Contact, concussion	0.336 [0.299, 0.345]	68.7 [65.6, 71.9]	0.198 [0.151, 0.290]	0.84 [0.55, 1.29]
Collision, no concussion	0.343 [0.323, 0.356]	65.2 [59.5, 69.0]	0.174 [0.156, 0.222]	0.78 [0.51, 1.02]
Collision, concussion	0.342 [0.329, 0.362]	65.0 [63.9, 68.5]	0.174 [0.135, 0.215]	0.84 [0.67, 1.43]

*Values are reported as median [minimum, maximum] for FA, MD, Gconn, and the ratio of NAA/Cr*.

*Significant group effects of concussion, based on bootstrapped mean differences.

## Discussion

This article presents a detailed comparison of white matter microstructure, resting brain function and cerebral metabolites, for athletes in sports associated with different levels of contact exposure. By imaging athletes prior to the start of their respective sporting season, the analyses focused on persistent markers of contact sport participation, limiting potential confounds associated with recent concussive and subconcussive impacts. The MRI data were analyzed using PLS, as this flexible multivariate model is sensitive to spatially distributed effects of contact exposure on the brain. For each MRI modality (DTI, fMRI, and SVS), the analyses showed significant variations between non-contact, contact, and collision sport participants providing a neuroimaging basis for the sport classifications defined by Meehan and colleagues (18).

The DTI analyses showed elevated FA and reduced MD for the collision sport group, whereas the non-contact and contact sport groups were overlapped. Thus, white matter microstructure appears to be fundamentally distinct among athletes that are exposed to routine, purposeful body-to-body collisions; this is supported by a previous DTI study in which cumulative effects of contact exposure were seen within a single season (13). The effects of contact exposure on DTI parameters are also consistent with the long-term effects of sport concussion, as increased FA and decreased MD have previously been reported (24–26), suggesting that both concussive and subconcussive impacts have similar effects on white matter microstructure. In this study, white matter regions where FA was related to contact exposure also showed significant effects of concussion history, whereas this was not observed for MD. This may be due to the concussion having more spatially sparse long-term effects on MD, as previously reported in Ref. (26). As the effects of contact exposure on FA and MD appear to be comparable in this study (i.e., the extent of significant brain regions and between-group differences), this suggests that MD may have more specificity for subconcussive contact exposure than FA. It is important to note that the trends in this study were opposite to those seen in more severe TBI (27–29), where decreased FA and increased MD are commonly reported markers of axonal injury. Thus, both concussion and participation in collision sports showed microstructural effects that appear to be distinct from the pathophysiology of more severe TBI. The cause of elevated FA and decreased MD in this cohort has not been definitively established, but may reflect adaptive growth processes in response to neural injury, e.g., axonal budding (30) or gliosis (31). Alternatively, these effects may be due to structural reorganization of white matter (32, 33), either to compensate for greater frequency of impacts, or in response to specific cognitive demands associated with collision sports. This is an important area of future research, where more advanced diffusion-weighted techniques may provide improved neurobiological specificity (34, 35).

The fMRI analyses showed a more gradual decrease in Gconn with greater contact exposure, as the intermediate contact group was overlapped with both non-contact and collision groups. The brain regions where Gconn was associated with contact exposure also showed significant effects of concussion history. These effects are consistent with previous studies of subacute concussion, where reduced connectivity has been observed (36, 37) and where a greater number of prior concussions was also associated with lower connectivity (36). In the present work, the observed connectivity changes were primarily seen in regions implicated in visual-motor function. The functioning of these brain regions is critical for sport performance, as well as avoiding injury during competition. Moreover, vulnerability of these domains to concussive and subconcussive impacts is supported by clinical literature, where visual-motor impairments are often observed following a concussion (38–40). Both Abbas et al. (14) and Johnson et al. (16) have previously examined subconcussive effects on the DMN, showing complex changes in network connectivity, both positive and negative. Consistent with these studies, no significant between-group differences were seen in Gconn for elements of the DMN, including the posterior cingulate, middle temporal lobes, and ventromedial prefrontal cortex (15), indicating the absence of a global shift in connectivity strength for this network (i.e., a uniform increase or decrease in connectivity). The decreased Gconn with greater contact exposure signifies reduced integrative brain function, which may stem from a combination of metabolic changes, altered cerebral blood flow, and injury to the neuroanatomical substrate (41, 42).

The analysis of cerebral metabolite ratios also showed consistent differences between sport groups. The only significant ratio was NAA/Cr, which decreased in sports associated with greater contact exposure. A decline in NAA has been previously observed in TBI patients (43, 44) and concussed athletes and may persist from days to months postinjury (45, 46), although non-significant findings have also been reported (47). The current findings suggest that the effect of participating in contact sports also exists along this spectrum. Although the specific function of NAA is disputed, it is thought to play a role in mitochondrial function and osmoregulation (48–50), and decreased NAA has been interpreted as a marker of neuronal loss (51) and/or reversible neurometabolic dysfunction (52). Cr is considered a stable reference peak for comparing metabolite ratios, due to the majority of Cr synthesis occurring external to the brain (53); therefore, the significant change of NAA relative to Cr is expected. The lack of significant group differences for Cho and Ins suggests that the primary effect of contact exposure is unlikely to be due to ongoing cellular degeneration or reactive gliosis, as Cho is usually implicated in cell membrane density, while Ins is implicated in glial cell proliferation (53). This further supports the decreases in NAA/Cr as an indicator of mainly neurometabolic dysfunction for athletes in contact and collision sports.

The present study describes a neurobiological “signature” of contact exposure in despite a lack of significant clinical impairments, which underscores the functional resilience of the brain, for active university-level athletes. Nonetheless, these results may provide a biological basis for the negative cognitive effects that have been reported with greater levels of contact exposure (9, 10), which may be driven by altered tissue microstructure and reduced neurometabolic function. The present findings also suggest persistent but potentially reversible effects of contact exposure, as white matter does not exhibit the reduced FA and elevated MD typically associated with more severe injury (27–29). In addition, decreased functional connectivity was associated with reduced NAA/Cr, indicating that the decreased functional integration may be driven by reduced neurometabolic activity (52). The observed physiological effects are highest for athletes in sports with greater contact exposure and with a history of concussion. An important question for future research is whether these MRI markers can be used to detect an “exposure threshold” to contact participation for which the brain cannot adapt, which may explain the negative long-term health consequences seen in a minority of athletes.

Although this study reported multiple neuroimaging markers that are consistently associated with participation in contact and collision sports, enhancing confidence that the findings are reliable, there are some limitations that should be considered for future research. One limitation is that the current study combined multiple different sports into contact and collision categories. Exposure to collisions may vary by sport within these categories, a hypothesis that is supported by epidemiological studies showing differences in concussion incidence across sports (54). Similarly, the prevalence and types of subconcussive impact may depend on the player position, playing style, and level of play; for example, Crisco et al. found that among collegiate football players, the frequency, location, and magnitude of impact vary with position (7, 55). Further research is required to determine how these factors influence neuroimaging markers. In addition, while the present study controlled for recent concussions, athletes may participate in preseason or off-season sports, leading to exposure to repetitive head impacts, which may confound the present findings. Nonetheless, the present results are encouraging, given the robust effects seen for a heterogeneous sample of different sports. At present, it cannot be determined to what degree the present results are driven by repeated subconcussive injury or by a previously undiagnosed concussion (56, 57). Future research should examine contact participation prospectively across the three different sport groups, using objective measures such as impact sensors and video monitoring to quantify the relationship between severity and frequency of impacts and neuroimaging biomarkers. Finally, future research should also examine how advanced MRI measures relate to other objective biomarkers. For example (58), reported significant correlations between participation in collision sports and expression of inflammatory blood biomarkers in healthy athletes. Integration with non-neuroimaging modalities will help to develop a more complete picture of the biological consequences of participating in contact and collision sports.

In summary, this study comprehensively examined neurobiological MRI markers in a group of healthy athletes sampled preseason. We identified robust effects on white matter microstructure, brain function, and neural metabolites in athletes who play sports with greater exposure to purposeful collisions. Our findings of MRI markers associated with contact exposure are qualitatively similar to the literature that has examined the measures in concussion, suggesting a continuum of changes even in the absence of diagnosed concussion, with greatest effects among athletes in collision sports. These findings provide important information about how MRI markers of brain health and concussion vary across athlete cohorts, improving our ability to model accurately the long-term effects of brain injury and exposure to repetitive impacts in sport.

## Ethics Statement

This study was carried out in accordance with the recommendations of the Canadian Tri-Council Policy Statement 2 (TCPS2) and the research ethics boards of the University of Toronto and St. Michael’s Hospital, with written informed consent from all subjects. All subjects gave written informed consent in accordance with the Declaration of Helsinki. The protocol was approved by the research ethics boards of the University of Toronto and St. Michael’s Hospital.

## Author Contributions

NC, MH, and TS conceptualized and planned the study. NC performed analyses and manuscript preparation. TS, MH, ADB, and SG revised for critical intellectual content and assisted with interpretation of findings.

## Conflict of Interest Statement

The authors declare that the research was conducted in the absence of any commercial or financial relationships that could be construed as a potential conflict of interest.

## Footnotes

^1^www.fmrib.ox.ac.uk/fsl.

^2^afni.nimh.nih.gov.

^3^nitrc.org/projects/spikecor.

^4^nitrc.org/projects/phycaa_plus.

^5^tarquin.sourceforge.net.
